# Combination fixed-dose β agonist and steroid inhaler as required for adults or children with mild asthma: a Cochrane systematic review

**DOI:** 10.1136/bmjebm-2021-111764

**Published:** 2021-07-19

**Authors:** Iain Crossingham, Sally Turner, Sanjay Ramakrishnan, Anastasia Fries, Matthew Gowell, Farhat Yasmin, Rebekah Richardson, Philip Webb, Emily O'Boyle, Timothy Stopford Christopher Hinks

**Affiliations:** 1 Respiratory Medicine, East Lancashire Hospitals NHS Trust, Blackburn, UK; 2 Respiratory Medicine Unit and National Institute for Health Research (NIHR) Oxford Biomedical Research Centre (BRC), Nuffield Department of Medicine, University of Oxford, Oxford, UK; 3 School of Medical and Health Sciences, Edith Cowan University, Joondalup, Western Australia, Australia; 4 New College, University of Oxford Medical School, Oxford, UK; 5 Pharmacy, Kettering General Hospital, Kettering, UK

**Keywords:** asthma, evidence-based practice, respiratory tract diseases, primary healthcare

## Abstract

**Background:**

In people with mild asthma poor adherence to regular therapy is common and increases the risk of exacerbations, morbidity and mortality. The use of fixed-dose combination inhalers containing an inhaled corticosteroid (ICS) and a fast-acting β_2_-agonist (FABA) is established in moderate asthma, but they may also have potential utility in mild asthma.

**Objectives:**

To evaluate the efficacy and safety of single combined FABA/ICS inhaler only used as needed in people with mild asthma.

**Design and setting:**

Cochrane meta-analysis of available trial data.

**Participants:**

Children aged 12+ and adults with mild asthma.

**Search methods:**

We searched the Cochrane Airways Trials Register, Cochrane Central Register of Controlled Trials, MEDLINE and Embase, ClinicalTrials.gov and the WHO trials portal on 19 March 2021.

**Interventions:**

A single fixed-dose FABA/ICS inhaler used as required compared with no treatment, placebo, short-acting beta agonist (SABA) as required, regular ICS with SABA as required, regular fixed-dose combination ICS/long-acting beta agonist (LABA), or regular fixed-dose combination ICS/FABA with as required ICS/FABA. We included randomised controlled trials (RCTs) and cross-over trial. We excluded trials shorter than 12 weeks. We included full texts, abstracts and unpublished data.

**Data collection and analysis:**

We used Cochrane’s standard methodological procedures and applied the GRADE approach to assess the evidence.

**Main outcome measures:**

We included six studies from which 9657 participants contributed to the meta-analyses. All used dry powder budesonide and formoterol as the combination inhaler. Two studies included children aged 12+ years and two studies were open-label.

**FABA/ICS as-required versus FABA as-required:**

Compared with as-required FABA alone, as-required FABA/ICS reduced exacerbations requiring systemic steroids (OR 0.45, 95% CI 0.34 to 0.60, 2 RCTs, 2997 participants, high-certainty evidence), equivalent to 109 people out of 1000 in the FABA alone group experiencing an exacerbation requiring systemic steroids, compared with 52 (95% CI 40 to 68) out of 1000 in the FABA/ICS as-required group. FABA/ICS as required may also reduce the odds of an asthma-related hospital admission or emergency department or urgent care visit (OR 0.35, 95% CI 0.20 to 0.60, 2 RCTs, 2997 participants, low-certainty evidence). Changes in asthma control were small and less than the minimal clinically important difference (MCID). FABA/ICS as required was associated with reductions in fractional exhaled nitric oxide, probably reducing the odds of an adverse event (OR 0.82, 95% CI 0.71 to 0.95) and may reduce total systemic steroid dose (mean difference (MD) −9.90, 95% CI −19.38 to −0.42).

**FABA/ICS as required versus regular ICS plus FABA as required:**

There may be little or no difference in the number of people with asthma exacerbations requiring systemic steroids with FABA/ICS as required compared with regular ICS (OR 0.79, 95% CI 0.59 to 1.07, 4 RCTs, 8065 participants, low-certainty evidence), equivalent to 81 people out of 1000 in the regular ICS plus FABA group experiencing an exacerbation requiring systemic steroids, compared with 65 (95% CI 49 to 86) out of 1000 in the FABA/ICS as-required group. The odds of an asthma-related hospital admission or emergency department or urgent care visit may be reduced in those taking FABA/ICS as required (OR 0.63, 95% CI 0.44 to 0.91, 4 RCTs, 8065 participants, low-certainty evidence). Changes in asthma control were small and less than MCID. Adverse events and total systemic corticosteroid doses were similar between groups. FABA/ICS as required was likely associated with less average daily exposure to ICS than those on regular ICS (MD −154.51 mcg/day, 95% CI −207.94 to −101.09).

**Conclusions:**

FABA/ICS as required is clinically effective in adults and adolescents with mild asthma and reduced exacerbations, hospital admissions or unscheduled healthcare visits and exposure to systemic corticosteroids and probably reduces adverse events compared with FABA as required alone. FABA/ICS as required is as effective as regular ICS and reduced asthma-related hospital admissions or unscheduled healthcare visits, and average exposure to ICS, and is unlikely associated with increased adverse events.

**Summary box**

**What is already known about this subject?**

- Poor adherence to inhaled corticosteroids (ICS) in mild asthma is associated with preventable exacerbations.

**What are the new findings?**

- This meta-analysis of five randomised controlled trials enrolling 9657 participants found symptom-driven, as-required use of fast-acting β_2_-agonist (FABA)/ICS compared with reliever-only treatment reduced severe exacerbations requiring tablet steroids and rates of emergency admission to hospital with asthma symptoms. Symptom-driven, as-required use of FABA/ICS compared with regular daily ICS led to similar rates of severe exacerbations but lower rates of hospital admission and lower total ICS dose.

**How might it impact clinical practice in the foreseeable future?**

- These findings support changes in guidelines away from the use of short-acting β_2_-agonists alone in mild asthma. Use of as-required FABA/ICS is a therapeutic alternative to maintenance ICS in mild asthma, associated with reduced hospital admissions for asthma and reduced average daily exposure to ICS.

## Background and objectives

Between 45% and 70% of the 350 million people worldwide living with asthma have mild disease,^1–3^ and yet continue to suffer intermittent severe asthma attacks requiring oral steroids or hospital admission, and in some cases leading to asthma-related deaths. Inhaled corticosteroids (ICS) are effective in achieving disease control and reducing mortality,^4^ but intermittence of symptoms in mild asthma often leads to poor inhaler adherence,^5^ with consequent risk of exacerbations. A new treatment approach being considered in mild asthma is the use of single combined (fast-acting β_2_-agonist (FABA) plus an ICS) inhaler only used as needed according to symptoms, which may increase adherence in those at greatest risk of exacerbations. This Cochrane Review aims to summarise the data on efficacy and safety of single combined FABA/ICS inhaler used only as needed in people with mild asthma, to guide clinicians and policy makers in decision making.

The mainstay of asthma therapy is treatment with inhaled FABA, typically taken as required to relieve bronchospasm, and ICS as regular preventive therapy. Although ICS are very effective in reducing severe asthma exacerbations and asthma deaths^4 6^ the intermittent nature of symptoms in mild asthma, the slower perceived response to ICS and concerns about steroid-related side effects frequently lead to poor adherence to regular ICS.^5 7^ In the UK the majority of asthma deaths occur in those considered to have mild or moderate asthma,^8^ with over-reliance on reliever medication^7^ and poor adherence to preventer ICS considered to be a main cause for an increase in risk of exacerbations in people with mild asthma.^9^

Fixed-dose combination inhalers containing both a steroid and a FABA in the same device, used as both maintenance and reliever therapy simplify inhaler regimens and ensure symptomatic relief is accompanied by preventative therapy. Their use is established in moderate asthma,^10^ but they may also have potential utility in mild asthma. Globally, prevalence of mild asthma is estimated to be between 45% and 70% of all patients diagnosed with asthma.^1 2^ We assessed the efficacy and safety of single combined FABA/ICS inhaler used only as needed in people with mild asthma. We compared this with two current approaches to treatment: use only of a FABA when needed, or use of a FABA when needed on top of regular-maintenance ICS.

Several clinical trials of as-required fixed-dose combination inhalers have been reported in recent years, and have led to a significant change in an international guideline,^11^ which now recommends fixed-dose ICS/FABA as first-line therapy for mild asthma, where the previous guideline recommended use of short-acting β agonist (SABA) only. We aimed to provide an objective, global review of the available evidence to inform decision makers, clinicians and people with asthma.

## Methods

We followed a published protocol in *The Cochrane Library*. We included randomised controlled trials (RCTs) and cross-over trials with at least a 1-week washout period. We included studies of a single fixed-dose FABA/ICS inhaler used as required compared with no treatment, placebo, SABA as required, regular ICS with SABA as required, regular fixed-dose combination ICS/long-acting β agonist, or regular fixed-dose combination ICS/FABA with as-required ICS/FABA. We planned to include cluster-randomised trials if the data had been or could be adjusted for clustering. We excluded trials shorter than 12 weeks. We included full texts, abstracts and unpublished data.

We included adults and children (age 6 years and older) with a diagnosis of mild asthma as defined by Global INitiative for Asthma (GINA) 2019.^11^ We excluded participants with chronic obstructive pulmonary disease or other respiratory comorbidity. We excluded participants receiving regular moderate or high-dose ICS, defined as ≥300 mcg per day of beclomethasone equivalent.

We assessed the following primary outcomes: 1. Exacerbations requiring systemic steroids; 2. Hospital admissions/emergency department or urgent care visits for asthma; 3. Measures of asthma control: in order of preference Asthma Control Questionnaire (ACQ), asthma control test, symptom-free days.

We assessed the following secondary outcomes: 1. Measures of lung physiology: in order of preference postbronchodilator; forced expieratroy volume in 1 second (FEV_1)_, postbronchodilator peak expiratory flow rate (PEFR), fractional exhaled nitric oxide (FeNO), then other measures; 2. Quality of life measures, preferably Asthma Quality of Life Questionnaire, then the Short Form 36. 3. Adverse events/side effects; 4. Total inhaled steroid dose; 5. Total systemic corticosteroid dose; 6. Mortality.

### Data collection and analysis

We searched the Cochrane Airways Trials Register, Cochrane Central Register of Controlled Trials, Medical Literature Analysis and Retrieval System Online (MEDLINE) and Embase, ClinicalTrials.gov and the WHO trials portal. We used Cochrane’s Screen4Me workflow to help assess search results and followed a prepublished protocol. Four authors screened titles and abstracts independently using Rayyan, with each abstract screened by at least two review authors. We contacted trial authors for further information and requested details regarding the possibility of unpublished trials. The most recent search was conducted on 19 March 2021. Two review authors independently extracted outcome data from included studies. Bias was assessed using V.5.1 of the Cochrane Handbook for Systematic Reviews of Interventions, assessing the following domains: 1. Random sequence generation, 2. Allocation concealment, 3. Blinding of participants and personnel, 4. Blinding of outcome assessment, 5. Incomplete outcome data, 6. Selective outcome reporting, 7. Other bias.

We analysed dichotomous data as ORs or rate ratios and continuous data as mean difference (MD). We reported 95% CIs. We used Cochrane’s standard methodological procedures of meta-analysis. We intended to assess publication bias. We applied the Grading of Recommendations Assessment, Development and Evaluation (GRADE) approach to summarise results and to assess the overall certainty of evidence.

## Main results

**Figure 1:**
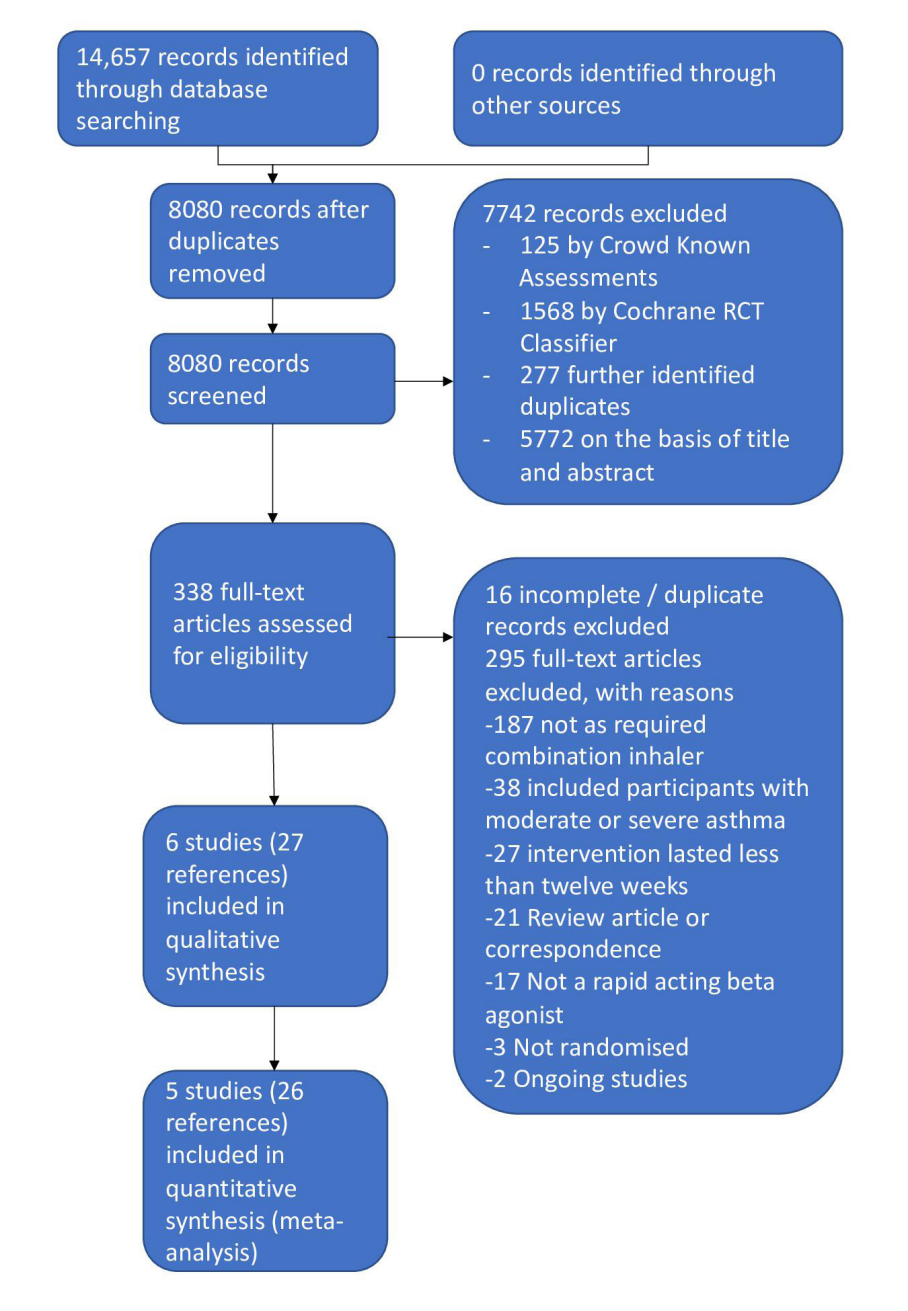
Preferred reporting items for systematic reviews and meta-analyses (PRISMA) flow diagram. RCT, randomised controlled trial.

We identified 14 657 records in our literature searches (figure 1). We included six studies of which five contributed results to the meta-analyses.^12–16^ Four studies were large, multinational studies from the same research group which used budesonide 200 mcg and formoterol 6 mcg in a dry powder formulation as the combination inhaler. Comparator fast-acting bronchodilators included terbutaline and formoterol. Two studies included children aged 12+ years and adults; two studies were open label. A total of 9657 participants was included, with a mean age of 36–43 years; 2.3%–11% were current smokers.

Two studies were open label (Novel START^13^ and PRACTICAL)^14^ and were judged as high risk of bias in this domain, but all studies were otherwise of low risk of bias in other domains. Four of the RCTs were funded by AstraZeneca.

### FABA/ICS as required versus FABA as required

**Figure 2:**
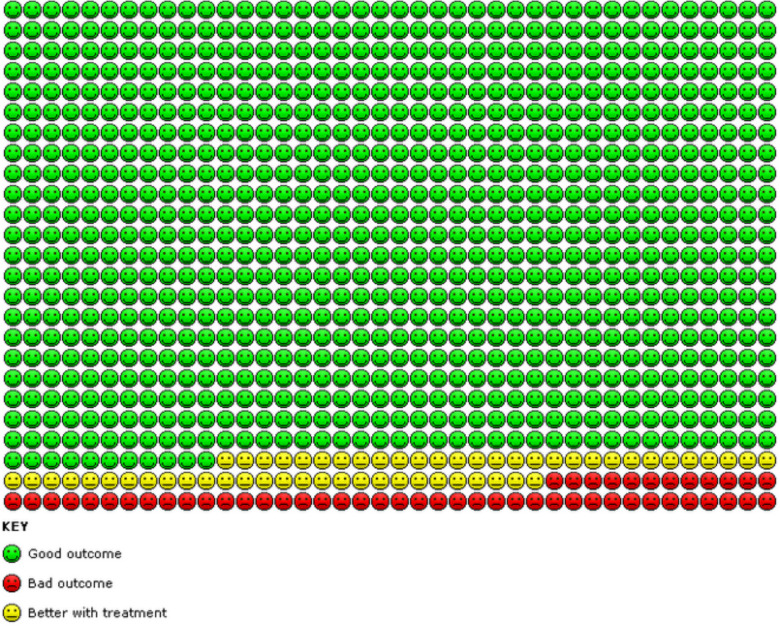
In the FABA as-required group, 109 people out of 1000 had exacerbations requiring systemic steroids over 52 weeks, compared with 52 (95% CI 40 to 68) out of 1000 in the FABA/ICS as-required group. FABA, fast-acting β_2_-agonist; ICS, inhaled corticosteroid.

Results for this comparison are presented in the Summary of findings 1 (table 1). We found evidence from two trials (Novel START,^13^ SYGMA 1)^15^ that compared with as-required β-agonists alone, as-required FABA/ICS significantly reduced the number of asthma exacerbations requiring systemic steroids over a 52-week period (OR 0.45, 95% CI 0.34 to 0.60; n=2997, high-certainty evidence). This is equivalent to 109 people out of 1000 in the FABA alone group experiencing an exacerbation requiring systemic steroids, compared with 52 (95% CI 40 to 68) out of 1000 in the FABA/ICS as-required group (represented graphically in figure 2).

**Table 1:** Summary of findings 1. As-required FABA/ICS inhalers compared with as-required FABA inhalers for mild asthma

As‐required FABA/ICS inhalers compared with as‐required FABA inhalers for mild asthma						
**Patient or population:** Mild asthma **Setting:** Community **Intervention:** As‐required FABA/ICS inhalers **Comparison:** As‐required FABA inhalers						
**Outcomes**	**Anticipated absolute effects^$^ (95% CI**)		**Relative effect** (**95% CI**)	**Number of participants** (**studies**)	**Certainty of the evidence** (**GRADE**)	**Comments**
	**Risk with as-required FABA inhalers**	**Risk with as-required FABA/ICS inhalers**				
Asthma exacerbation requiring systemic steroid follow‐up: 52 weeks	109 per 1000	52 per 1000 (40 to 68)	OR 0.45, 95% CI 0.34 to 0.60	2997 (2 RCTs)	⊕⊕⊕⊕ HIGH*†	People with mild asthma treated with combined inhalers have substantially fewer exacerbations requiring systemic steroid than those treated with FABA alone.
Hospital admission, ED and urgent care visits follow‐up: 52 weeks	34 per 1000	12 per 1000 (7 to 21)	OR 0.35, 95% CI 0.20 to 0.60	2997 (2 RCTs)	⊕⊕⊝⊝ LOW†‡	People with mild asthma treated with combined inhalers probably have substantially fewer exacerbations requiring hospital admission, ED attendance or urgent care visit than those treated with FABA alone.
Asthma control follow‐up: 52 weeks Lower scores=better control	Mean baseline ACQ‐5 ranged from 1.1 to 1.61	MD −0.15, 95% CI −0.20 to −0.10	‐	2859 (2 RCTs)	⊕⊕⊕⊝ MODERATE†	MCID for ACQ‐5 is 0.5. A third study reported no difference in asthma symptom scores between the two arms.
Inhaled steroid dose assessed with: mean daily inhaled steroid dose, μg beclomethasone equivalent follow‐up: 52 weeks	The mean inhaled steroid dose was 18.7 µg beclomethasone	MD 76.50 µg beclomethasone higher (69.40 higher to 83.60 higher)	‐	2554 (2 RCTs)	⊕⊕⊕⊝ MODERATE†	People with mild asthma treated with a combined inhaler have a higher daily inhaled steroid dose than those treated with a FABA alone.
Total systemic steroid dose assessed with: mg prednisolone total over 52 weeks follow‐up: 52 weeks	The mean total systemic steroid dose was 17.4 mg prednisolone	MD 9.90 mg prednisolone lower (19.38 lower to 0.42 lower; participants=443)	‐	443 (1 RCT)	⊕⊕⊝⊝ LOW†§	Total systemic steroid dose was similar and small in both: those given combined inhalers and those given FABA alone.
Adverse events follow‐up: 52 weeks	486 per 1000	437 per 1000 (402 to 473)	OR 0.82 (0.71 to 0.95)	3002 (2 RCTs)	⊕⊕⊕⊝ MODERATE†	Slightly fewer adverse events occurred in those taking combination inhalers compared with those taking FABA alone.
**^$^The risk in the intervention group** (and its 95% CI) is based on the assumed risk in the comparison group and the **relative effect** of the intervention (and its 95% CI).						
**GRADE Working Group grades of evidence** **High certainty** ⊕⊕⊕⊕: we are very confident that the true effect lies close to that of the estimate of the effect. **Moderate certainty ⊕⊕⊕:** we are moderately confident in the effect estimate; the true effect is likely to be close to the estimate of the effect, but there is a possibility that it is substantially different. **Low certainty ⊕⊕:** our confidence in the effect estimate is limited; the true effect may be substantially different from the estimate of the effect. **Very low certainty ⊕:** we have very little confidence in the effect estimate; the true effect is likely to be substantially different from the estimate of effect.						

*Upgraded as large effect (OR <0.5) with fairly tight CIs.

†Downgraded as included open label study.

‡Downgraded as based on a small number of events.

§Downgraded as based on one study with a relatively small number of participants.

ACQ-5, Asthma Control Questionnaire‐5; ED, emergency department; FABA, fast‐acting β₂‐agonist; ICS, inhaled corticosteroid; MCID, minimum clinically important difference; MD, mean difference; RCT, randomised controlled trial.

We found a reduction in the odds of hospital admission or emergency department or urgent care visit for asthma in participants given as-required FABA/ICS compared with as-required FABA alone (OR 0.35, 95% CI 0.20 to 0.60; n=2997, low-certainty evidence).

Compared with as-required FABA alone, any changes in asthma control or spirometry, though favouring as-required FABA/ICS, were small and less than the minimal clinically important differences (MCIDs). We did not find evidence of differences in asthma-associated quality of life or mortality. For other secondary outcomes FABA/ICS as required was associated with reductions in FeNO, probably reducing the odds of an adverse event (OR 0.82, 95% CI 0.71 to 0.95, 2 RCTs, 3002 participants, moderate-certainty evidence) and may reduce total systemic steroid dose (MD −9.90, 95% CI −19.38 to −0.42, 1 RCT, 443 participants, low-certainty evidence), with an increase in the daily inhaled steroid dose (MD 77 mcg beclomethasone equivalent/day, 95% CI 69 to 84, 2 RCTs, 2554 participants, moderate-certainty evidence).

We did not find a clear difference in serious adverse events in the three trials reporting this outcome, though CIs were wide (OR 1.31, 95% CI 0.50 to 3.46; n*=*3095). In the two trials reporting all adverse events, the odds of an adverse event were 18% lower in the as-required FABA/ICS group compared with the as-required FABA group (OR 0.82, 95% CI 0.71 to 0.95; n=3002, moderate-certainty evidence).

There was no difference in mortality observed, but this was based on a single death in the three studies, so no conclusions could be drawn about mortality differences.

### FABA/ICS as required versus regular ICS plus FABA as required

**Figure 3:**
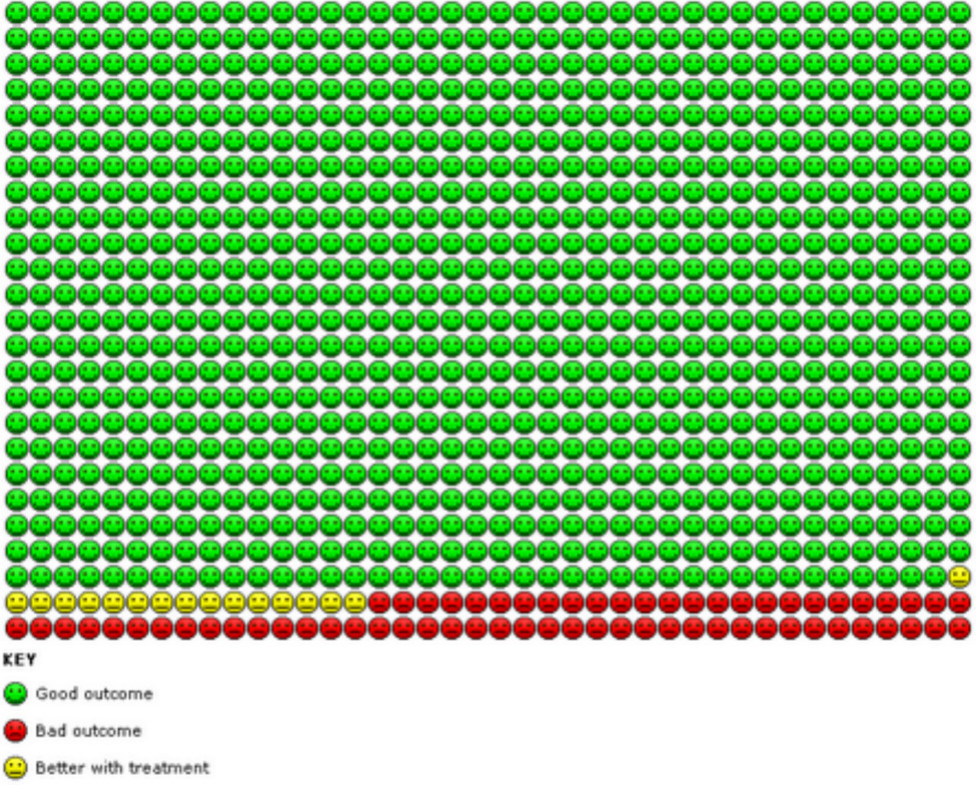
In the regular ICS group 81 people out of 1000 had exacerbations requiring systemic steroids over 52 weeks, compared with 65 (95% CI 49 to 86) out of 1000 in the FABA/ICS as-required group. FABA, fast-acting β_2_-agonist; ICS inhaled corticosteroid.

Results for this comparison are presented in the Summary of findings (table 2). We found evidence based on four studies^13–16^ that the odds of an asthma exacerbation requiring systemic steroids were reduced in participants treated with as-required FABA/ICS compared with regular ICS, but CIs include no difference (OR 0.79, 95% CI 0.59 to 1.07; n=8065, low-certainty evidence). This is equivalent to 81 people out of 1000 in the regular ICS plus FABA group experiencing an exacerbation requiring systemic steroids, compared with 65 (95% CI 49 to 86) out of 1000 in the FABA/ICS as-required group (represented graphically in figure 3). There were fewer exacerbations of asthma requiring either hospital admission or a visit to an emergency department or urgent care clinic in participants taking as-required FABA/ICS compared with regular ICS (OR 0.63, 95% CI 0.44 to 0.91; n=8065, low-certainty evidence).

**Table 2:** Summary of findings 2. As-required FABA/ICS inhalers compared with regular inhaled steroids for mild asthma

As‐required FABA/ICS inhalers compared with regular inhaled steroid for mild asthma						
**Patient or population:** Mild asthma **Setting:** Community **Intervention:** As‐required FABA/ICS inhalers **Comparison:** Regular inhaled steroid						
**Outcomes**	**Anticipated absolute effects^$^ (95% CI**)		**Relative effect** (**95% CI**)	**Number of participants** (**studies**)	**Certainty of the evidence** (**GRADE**)	**Comments**
	**Risk with regular inhaled steroid**	**Risk with as-required FABA/ICS inhalers**				
Exacerbations requiring systemic steroid follow‐up: 52 weeks	81 per 1000	65 per 1000 (49 to 86)	OR 0.79 (0.59 to 1.07)	8065 (4 RCTs)	⊕⊕⊝⊝ LOW*†	Exacerbations requiring systemic steroid occurred less frequently in those treated with as-required combination inhalers than those treated with regular inhaled steroids, but the 95% CI includes no difference.
Hospital admission, ED and urgent care visits follow‐up: 52 weeks	19 per 1000	12 per 1000 (8 to 17)	OR 0.63 (0.44 to 0.91)	8065 (4 RCTs)	⊕⊕⊝⊝ LOW*‡	Fewer hospital admissions, ED attendances and urgent care visits occurred in those treated with as-required combination inhalers compared with regular inhaled steroids.
Asthma control assessed with: ACQ‐5, follow‐up: 52 weeks. Lower scores indicate better asthma control	The mean asthma control was −0.467 points, change from baseline	MD 0.12 points higher (0.09 higher to 0.15 higher)	‐	7382 (4 RCTs)	⊕⊕⊕⊕ HIGH	ACQ‐5 fell slightly more compared with baseline in those treated with regular inhaled steroids than those treated with combination inhalers. MCID for ACQ‐5 is 0.5 points.
Inhaled steroid dose assessed with: mean daily dose in μg, beclomethasone equivalent follow‐up: 52 weeks	The mean inhaled steroid dose was 257.8 µg beclomethasone equivalent per day	MD 154.51 µg/day lower (207.94 lower to 101.09 lower)	‐	7180 (4 RCTs)	⊕⊕⊕⊝ MODERATE*	Those treated with as-required combination inhalers had a lower average daily inhaled steroid dose than those treated with a regular inhaled steroid.
Total systemic steroid dose assessed with: mean cumulative dose of prednisolone over the course of the trial in mg follow-up: 52 weeks	The mean total systemic steroid dose was 20.97 mg prednisolone	MD 7 mg prednisolone lower (13.97 lower to 0.03 lower)	‐	1330 (2 RCTs)	⊕⊕⊕⊝ MODERATE*	Total systemic steroid exposure was similar and low in those treated with regular inhaled steroid and those treated with as-required combination inhalers.
Adverse events assessed with: Participants experiencing at least one adverse event follow‐up: 52 weeks	493 per 1000	482 per 1000 (443 to 525)	OR 0.96 (0.82 to 1.14)	8072 (4 RCTs)	⊕⊕⊕⊝ MODERATE*	The proportion of participants experiencing at least one adverse event was similar in those treated with combination inhalers and those with regular inhaled steroid.
^$^ **The risk in the intervention group** (and its 95% CI) is based on the assumed risk in the comparison group and the **relative effect** of the intervention (and its 95% CI).						
**GRADE Working Group grades of evidence** **High certainty ⊕⊕⊕⊕:** we are very confident that the true effect lies close to that of the estimate of the effect. **Moderate certainty ⊕⊕⊕:** we are moderately confident in the effect estimate; the true effect is likely to be close to the estimate of the effect, but there is a possibility that it is substantially different. **Low certainty ⊕⊕:** our confidence in the effect estimate is limited; the true effect may be substantially different from the estimate of the effect. **Very low certainty ⊕:** we have very little confidence in the effect estimate; the true effect is likely to be substantially different from the estimate of effect.						

*Downgraded as included open label studies.

†Downgraded as heterogeneity between trials at low risk of bias in all domains and those at high risk in at least one domain.

‡Downgraded as based on a relatively small number of events.

ACS-5, Asthma Control Questionnaire‐5; ED, emergency department; FABA, fast‐acting β₂‐agonist; ICS, inhaled corticosteroid; MCID, minimum clinically important difference; MD, mean difference; RCT, randomised controlled trial.

When assessing ACQ-5 data we found a statistical advantage to regular ICS compared with as-required FABA/ICS but the absolute differences were small (MD 0.12, 95% CI 0.09 to 0.15; participants=7382) and less than the MCID, which for ACQ-5 is 0.5 points. Compared with regular ICS, any changes in asthma control, spirometry, peak flow rates or asthma-associated quality of life, though favouring regular ICS, were small and less than the MCIDs. Adverse events, serious adverse events, total systemic corticosteroid dose and mortality were similar between groups, although deaths were rare, so CIs for this analysis were wide. We found moderate-certainty evidence from four trials involving 7180 participants that FABA/ICS as required was likely associated with less average daily exposure to ICS than regular ICS (MD −154.51 mcg/day, 95% CI −207.94 to −101.09).

In preplanned sensitivity analyses, excluding the two open-label studies^13 14^ did not alter the direction of effect in any of the primary outcomes, neither did use of a fixed-effects rather than a random-effects model.

## Discussion and conclusions

We found moderate-certainty evidence to high-certainty evidence that as-required fixed-dose FABA/ICS is clinically effective in adults and adolescents with mild asthma. Their use instead of FABA as required alone reduced exacerbations, hospital admissions or unscheduled healthcare visits, and exposure to systemic corticosteroids, and probably reduced adverse events. As exacerbations are responsible for the majority of morbidity, mortality and the economic costs of asthma, this would support recent changes in international guidelines (GINA 2019) away from the use of SABA alone in mild asthma.

Furthermore, use of as-required FABA/ICS is as effective as regular ICS, and is associated with a reduction of asthma-related hospital admissions or unscheduled healthcare visits, while reducing the average daily exposure to ICS, without any evidence of an increase in adverse events. As mild asthma is heterogeneous, with eosinophilic airway inflammation— linked to the greatest benefit from ICS—present in only a quarter of individuals,^17^ these dual benefits are likely to be achieved by a symptom-driven therapy reducing intentional and unintentional use of FABA in monotherapy in these individuals. We now recognise distinct asthma phenotypes.^18^ Those with type-2 high disease have steroid-responsive inflammation associated with high peripheral blood and sputum eosinophil counts, and high FeNO, and are at risk of exacerbations. As it is hard to distinguish between type-2 high and type-2 low disease in primary care, the symptom-driven approach effectively enables those with symptomatic type-2 high disease to self-titrate their therapy in line with the level of underlying steroid-responsive inflammation.

We believe these studies are representative of adults with mild asthma in the real world, with broad inclusion criteria, with only two of the studies that contributed data requiring reversibility as an inclusion criterion, the others depending on self-report of physician-diagnosed asthma. Participants had mean age 36–43 years, a mild deficit in baseline lung function (prebronchodilator FEV_1_ 84%–90%) and included current smokers (2.3%–11% of participants), and those with a range of preceding annual exacerbation rates (5.5%–22%). These results are therefore likely to be generalisable to populations with mild asthma in primary care.

Using the GRADE system, we judged the certainty of the evidence per outcome for main comparisons—those related to rates of exacerbations—to be low (with the exception of exacerbations requiring systemic steroid in the as-required FABA/ICS vs as-required FABA comparison). This judgement may be overly conservative, as the results are based solely on relevant, well-designed RCTs. The methodological quality was otherwise good for the included trials; they were conducted in applicable populations, examining outcomes of direct relevance to participants, with low–moderate heterogeneity across studies, and with consistent findings between studies, including between blinded and unblinded studies.

Our findings are consistent with data from a 2007 double-blind RCT which showed as-required beclomethasone-salbutamol 250/100 mcg in a single inhaler was as effective as regular use of inhaled beclomethasone 250 mcg twice daily and more effective than as-required salbutamol alone in preventing exacerbations and improving morning PEFR.^19^ That study was judged as at low risk of bias, but was excluded because 31.6% of participants were receiving regular ICS, with a mean dose of 460 mcg/day.

These findings support as-required use of FABA/ICS in a fixed dose combination inhaler as superior to SABA alone, and as a therapeutic alternative to maintenance ICS in mild asthma, could reduce the number of severe asthma attacks. This is important as asthma is a major cause of time off work, economic costs and chronic ill-health, and it remains a largely preventable cause of death for 400 000 people per year globally. This approach also simplifies treatment regimens and could reduce contradictory or ambiguous messages to people with asthma. It would support adoption of this strategy in current guidelines. However, cost frequently limits the availability of these inhalers in many low-income and middle-income countries, who rely heavily on reliever therapies or tablet steroids instead. Further pragmatic studies and healthcare cost assessments in such countries are needed to support equitable access to affordable quality-assured asthma medicines.

The implementation of these findings may depend on differing health economic assessments, differing healthcare infrastructures and population-specific factors in different settings globally. All the data are derived from studies of dry powder formulations and may not necessarily apply to pressurised metred-dose inhalers. Further research is needed to explore use of FABA/ICS as required in children under 12 years of age, use of other FABA/ICS preparations, health economic factors and long-term outcomes beyond 52 weeks.

## Data Availability

Data sharing is not applicable as no data sets were generated and/or analysed for this study.
